# The Treatment of Suppurating Glands of the Neck

**Published:** 1871-04

**Authors:** 


### THE TREATMENT OF SUPPURATING GLANDS OF THE NECK.

Dr. John Murray says in *The British Medical Journal:*

The interesting communication by Mr. Lawson Tait, on this subject, in which he recommends frequent tapping, leads me to suggest that more consideration should be shown by practitioners to the simple plan of drainage than is, I believe, the custom.

A case at present under my care is sufficiently illustrative of the method and of the remarks which I wish to make. A girl, eleven years of age, presented herself on January 16th at the Middlesex Hospital, with several enlarged cervical glands, one of which, as large as a chestnut, had already suppurated. I passed at once two silk ligatures through the tumor by means of a needle, and tied the free ends. The mother was directed to keep the openings patent by moving the ligatures a little twice or thrice a day. In a week the tumor had much diminished, a considerable quantity of pus having been discharged. I then introduced, in place of the silk sutures, one of Professor Lister's cat-gut ligatures, soaked in carbolic oil, which answered the purpose admirably. This was removed in three or four days. On the 1st of February the abscess had ceased discharging, and the needle wounds had healed. There were still, however, some tenderness and redness over the seat of the abscess, with induration of the skin, which are, however, now rapidly disappearing. The part is at present being painted with collodium flexile. The other gland enlargements has subsided. Unless something untoward happen, the child will be saved the disfigurement of the extensive and permanent scar which would have resulted from the ordinary treatment by incision; I say ordinary, because Professor Lister's results under his method are said to be excellent, little or no noticeable mark being left. I have myself exceedingly little experience of the drainage plan of treatment; it is popular with some, and as unpopular with others who have tried it; but that its success is insured, with very excellent results, the above case proves.

